# The Function of Ion Channels and Membrane Potential in Red Blood Cells: Toward a Systematic Analysis of the Erythroid Channelome

**DOI:** 10.3389/fphys.2022.824478

**Published:** 2022-02-01

**Authors:** Marieke von Lindern, Stéphane Egée, Paola Bianchi, Lars Kaestner

**Affiliations:** ^1^Sanquin Research and Landsteiner Laboratory, Department of Hematopoiesis, Amsterdam UMC, University of Amsterdam, Amsterdam, Netherlands; ^2^Department of Cell Biology and Genetics, Erasmus University Medical Center, Rotterdam, Netherlands; ^3^Integrative Biology of Marine Models, Station Biologique de Roscoff, CNRS, UMR 8227, Sorbonne Université, Roscoff Cedex, France; ^4^Laboratoire d’Excellence GR-Ex, Paris, France; ^5^Pathophysiology of Anemia Unit, Hematology Unit, Fondazione IRCCS Ca’ Granda Ospedale Maggiore Policlinico of Milan, Milan, Italy; ^6^Theoretical Medicine and Biosciences, Medical Faculty, Saarland University, Homburg, Germany; ^7^Dynamics of Fluids, Experimental Physics, Saarland University, Saarbrücken, Germany

**Keywords:** erythrocyte, erythropoiesis, patch-clamp, ion channel, electrophysiology, channelopathy, genotype-phenotype correlation, pseudo action potential

## Abstract

Erythrocytes represent at least 60% of all cells in the human body. During circulation, they experience a huge variety of physical and chemical stimulations, such as pressure, shear stress, hormones or osmolarity changes. These signals are translated into cellular responses through ion channels that modulate erythrocyte function. Ion channels in erythrocytes are only recently recognized as utmost important players in physiology and pathophysiology. Despite this awareness, their signaling, interactions and concerted regulation, such as the generation and effects of “pseudo action potentials”, remain elusive. We propose a systematic, conjoined approach using molecular biology, *in vitro* erythropoiesis, state-of-the-art electrophysiological techniques, and channelopathy patient samples to decipher the role of ion channel functions in health and disease. We need to overcome challenges such as the heterogeneity of the cell population (120 days lifespan without protein renewal) or the access to large cohorts of patients. Thereto we will use genetic manipulation of progenitors, cell differentiation into erythrocytes, and statistically efficient electrophysiological recordings of ion channel activity.

## Introduction

Ion channels are crucial to feel, to see, to hear, to move, and to think. In other words, they are essential to live. They are important membrane transporters that have the singular capacity to generate transmembrane currents, and to convey signals that rapidly modify the properties of a cell on a time scale of the order of μ-seconds. Their function is evident in sensory organs, including the skin, and excitable cells such as neurons, where they are the core of signal transduction. Remarkably, evolution has conserved ion channels in all cell types, also in red blood cells (RBCs). This may not be surprising because ion channels are important to sense shear stress, so called mechano-sensors. RBCs have to “feel” shear stress when they pass the capillary bed in the tissues, and during quality control when they pass the spleen and have to slide through slits between endothelial cells. The 2021 Nobel prize for medicine or physiology placed the mechano-sensor Piezo1, a nonspecific cation channel, in the spotlights.

Although RBCs were among the first cell types used to study ion channels with the patch-clamp technique (Hamill, 1981, 1983), researcher quickly lost interest in the topic, i.e., after a number of initial publications in the first half of the 1980s, for 15 years almost no work about RBC ion channels was published. Indeed, especially human RBC presented a number of bottlenecks such as the limited copy number of channels per cell (Grygorczyk et al., 1984; Egée et al., 2002; Bennekou et al., 2003; Kaestner, 2015) and the impossibility to experimentally manipulate them genetically which made their study in this aspect complex and difficult. Therefore, most of the information available today is scattered, contradictory and does not permit the reconstruction of the whole jigsaw puzzle that would reveal the role of ion channels in the function of the RBCs. This holds true, especially in the context that ion channels in RBCs are still being discovered, like the transient receptor potential channel TRPV2 (Belkacemi et al., 2021; Egee and Kaestner, 2021) suggesting that further ion channels can be discovered, or new electrophysiological effects like “pseudo action potentials,” which are found in RBCs (Jansen et al., 2021) and awaiting their full physiological explanation.

## Functional Evidence for Ion Channels in RBCs

There is a growing body of evidence that ion channels play an essential role in RBCs from their terminal differentiation in the bone marrow (Moura et al., 2019; Caulier et al., 2020), during their enucleation, and during the subsequent 120 days that they circulate in the bloodstream (Moras et al., 2017) and pass through capillaries sometimes much smaller than their own diameters (Faucherre et al., 2013; Cahalan et al., 2015; Danielczok et al., 2017). Mature RBCs cannot renew their proteins. Therefore, ion channels have to function or gradually lose their function within the 120 days (in humans) until the cells are removed from the circulation in the spleen (Deplaine et al., 2011). This generates a tremendous functional heterogeneity (Wang et al., 2013; Bogdanova et al., 2020).

In RBCs, activation of Piezo1 results in Ca^2+^ influx, which activates the Gárdos channel to release K^+^ from the cells and subsequently results in export of H_2_O. This rapid, transient process facilitates the cells to move through narrow passages. Congenital mutations in Piezo1 and the Gárdos channel were found in hemolytic anemia and unexpected iron overload, underscoring the importance of this ion channel interaction in RBCs. The role of most other ion channels remains unexplored.

## Technological Progress and Advancements of the Recent Years

Limitations and technological bottlenecks that have hitherto hindered the study of ion channels in RBCs are just about to be overcome: RBCs can be analyzed by automated high throughput patch-clamp robots (Rotordam et al., 2019), *in vitro* erythropoiesis is mastered up to mature erythrocytes (Heshusius et al., 2019) and available for systematic genetic screens (Verhagen et al., 2021), and significant cohorts of genotyped/phenotyped patients with channelopathies are available.

Transcriptomic (An et al., 2014) and proteomic (Gautier et al., 2016; Karayel et al., 2020) studies, clearly show that ion channels are not only differentially expressed upon differentiation but also selectively sorted during the enucleation step before the release of reticulocytes within the bloodstream. RBC production is qualitatively impaired in hereditary hemolytic anemia, linked directly or indirectly to ion channel defects (Moura et al., 2019; Caulier et al., 2020). This suggests that ion channels are critical regulators during the 2-week period required for complete maturation of a precursor culture to enucleated, hemoglobinized RBCs and for their function in the circulation. An accurate knowledge of the role of these channels is paramount for our understanding of erythropoiesis, especially as no less than 80 genes that are directly linked to membrane transport are expressed during erythropoiesis, including at least a dozen ion channels (Karayel et al., 2020). Tackling this knowledge gap will result in a major advance in the understanding and personalized management of rare inherited diseases but also in the role played by transport defects in much more common diseases such as sickle cell disease.

## RBC Ion Channels in Molecular Signaling

Enucleated RBCs have evolved to optimize the transport of O_2_ and CO_2_. These cells are unique as they have a membrane potential dictated by anions (in contrast to almost all other eukaryotic cells) to ensure optimal CO_2_ transport (V_M_ = −12 mV; Thomas et al., 2011). Consequently, RBCs must maintain their cationic permeability (Na^+^, K^+^, and Ca^2+^) at the lowest possible level to survive. Having a permeability tightly under control can be seen as a major advantage in terms of signaling because the simple flux of a few cations generates a variation in membrane potential equivalent to the variations recorded during a neuronal action potential (Dyrda et al., 2010; Jansen et al., 2021). The role of these “pseudo action potentials” occurring in RBCs is completely unknown. Importantly, ion channel activity becomes a threat if the fine-tuned equilibrium is disrupted by a genetic defect (Lew et al., 2002; Bogdanova et al., 2013; Rapetti-Mauss et al., 2017) or any other external signal (Li et al., 1996; Kaestner et al., 2004; Föller et al., 2008; Bogdanova et al., 2013; Wang et al., 2021). The dissipative power of the ion channels will immediately compromise the survival of the RBCs by altering their volume, shape, and deformability, which are the hallmarks of the longevity of RBCs within the circulation. The last decade has been marked by the discovery of several causative genes for hereditary RBC diseases (*PIEZO1*, Zarychanski et al., 2012, *KCNN4*, Glogowska et al., 2015; Rapetti-Mauss et al., 2015, and *RhAG*, Stewart et al., 2011). Their mutation alters the ionic balance directly (Rapetti-Mauss et al., 2015; Fermo et al., 2017, 2020; Hertz et al., 2017; Filser et al., 2020; Pérès et al., 2021) or indirectly as the result of deregulated ion channel activity (Barneaud-Rocca et al., 2011; Flatt et al., 2011). It has been hypothesized that incidence of this group of disorders may be underestimated and could affect one in 8,000 adults (Kaufman et al., 2018).

## Required Technological and Organizational Innovations

To obtain a better and more complete picture of the role of ion channels in RBCs, further efforts are required. Here, we propose two complementary concepts that need to be combined: (I) high throughput measurement of ion channel activity and membrane potential, and (II) the *in vitro* culture and maturation of genetically manipulated RBCs.

I: Automated patch-clamp technology became available to scientific community within the past 10–15 years. Due to the intrinsic properties of RBCs to pass small constrictions, e.g., capillaries or slits in the spleen, make them difficult to patch, i.e., seal them tightly on a small artificial opening (giga-seal formation). What is well described and required a lot of initial research in manual patch-clamp (Kaestner, 2011), holds also true for automated approaches, although, the aperture geometry changed from micropipettes to mainly planar chips with a hole. Therefore protocols to measure ion conductance and membrane potential of RBCs in automated high throughput patch-clamp robots need the active collaboration of the manufacturers to optimize chip geometries for RBCs (Rotordam et al., 2019). However, such a patch-clamp robot is to our best knowledge not available in any hematological laboratory – worldwide, i.e., the technology is in principle available but cannot be used for regular RBC research. Part of the problem is the high costs of initial required investment, the still unique qualification required to run the machines and in this context the putative operating grade, which might not be high enough to justify such an investment. Furthermore, hard core electrophysiology and hard core cell biology laboratories are often spatially separated (different Universities, even different countries). Shipment of RBCs is problematic and likely to result in compromised measurements (Makhro et al., 2016; Hertz et al., 2017; Rotordam et al., 2019). Therefore, we propose a mobile concept of such a patch-clamp robot. Such an implementation is not just placing a machine in a van, it is more like a small version of a space-lab module with a high integration level between the vehicle and the delicate instrument as well as the associated equipment to work autonomously from an independent power supply to sample preparation equipment. Nevertheless, Nan]i[on Technologies regards it a feasible concept (Nils Fertig and Michael George, personal communication). In the following, we refer to this mobile patch-clamp robot concept as the “PatchC]l[amper.”

II: Furthermore, a systematic approach of RBC investigations combining functional electrophysiology with a genetic approach based on an extended scale patient screening as well as accompanying *in vitro* erythropoiesis is necessary. This would allow to implement various concepts of genetic manipulations to functionalize RBCs and to manipulate their properties for medical needs (knock-out or downregulation of ion channels, their mutations – including the results from patients screening – the overexpression of ion channels to address experimental difficulties due to their low copy number in the membrane or even the expression of new and heterologous channels). The *in vitro* erythropoiesis also allows RBC age synchronization overcoming the RBC age variation occurring in human samples.

## Proposed Concrete Research Activities

**Figure 1:**
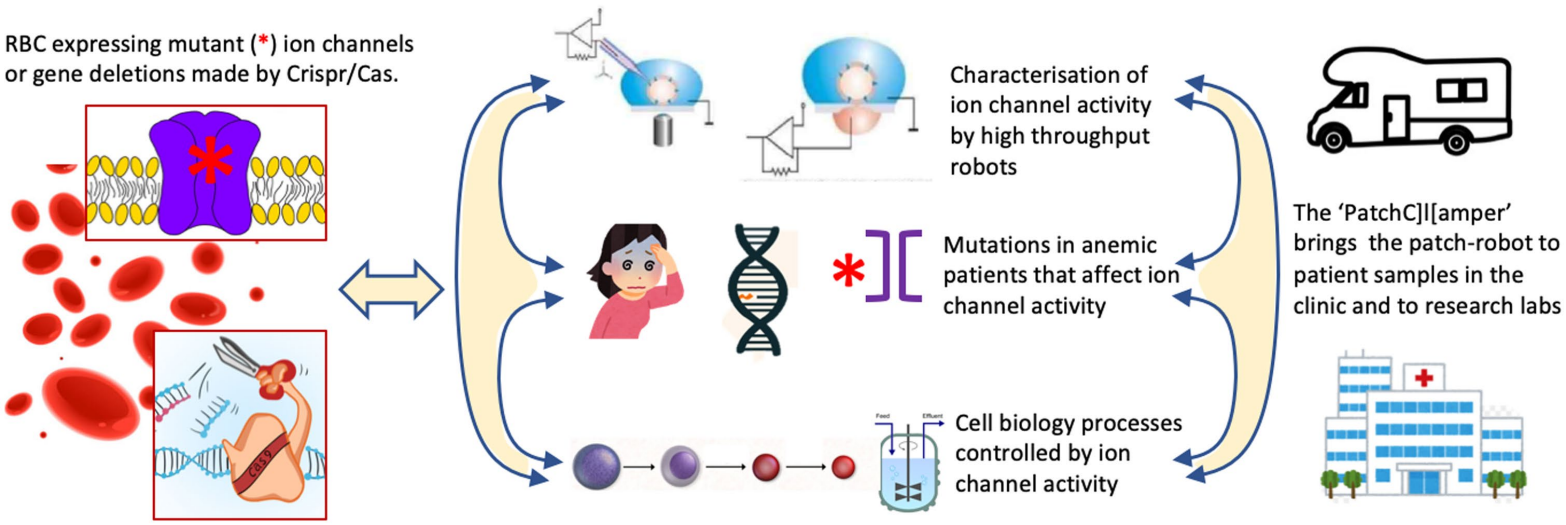
The proposed research activities to address the most urgent needs in red blood cell (RBC) ion channel research. One should make use of freshly isolated or cultured RBCs from healthy donors or channelopathy patients. Cultured precursors can be genetically modified to delete ion channels or to (over)express (mutated) ion channels in mature RBCs. This is the common basis for three conceptual research line activities. (i) The systematic investigation of RBC ion channels, their interactions, modulation by external factors and the induced signaling in the RBCs using classical patch-clamp and high throughput planer chips. (ii) Anemic patients suspected of channelopathies can be diagnosed by next generation sequencing (NGS) technology. This will allow to elucidate the molecular regulation of RBC channelopathies and RBC diseases with secondary involvement of ion channels. It should aid to develop more precise diagnosis and assessment of disease state, and future (personalized) therapeutic approaches. (iii) Although erythropoiesis is well documented for gene expression the functional activity of ion channels during the differentiation process needs to be investigated. Additionally, various gene-manipulation concepts should be applied and investigated in terms of cell function and efficiency of *in vitro* RBC production including the use of bioreactors.

The research innovations outlined above would require to be embedded into research activities to address the most urgent needs in RBC ion channel research. We propose three lines of activity as outlined below and summarized in Figure 1.

### To Understand the Regulation and Concerted Activity of Ion Channels in RBCs and to Unravel the Generation and Role of “Pseudo Action Potentials”

Based on the low K^+^ conductance of the RBC membrane, activations of the Gárdos channel (K_Ca_3.1) result in a hyperpolarization of the membrane potential (Monedero Alonso et al., 2021). Because the Gárdos channel number per RBC is low (in average approximately 10 copies per cell) and due to the stochastic nature of the channel openings, membrane potential jumps (cycles of hyperpolarization and depolarizations) occur, an effect that we call “pseudo action potential”. These “pseudo action potentials” appear to induce the opening of voltage activated Ca^2+^ channels in RBCs (Kaestner et al., 2018; Jansen et al., 2021). This process could partly explain the increased intracellular Ca^2+^ in patients with gain of function mutations of the Gárdos channel (Fermo et al., 2017, 2020; Jansen et al., 2021). This “pseudo action potential” is a new and unique finding among non-excitable cells and requires further investigations concerning its contribution to the ion homeostasis, the elucidation of the associated Ca^2+^ signaling and the involvement of other ion channels as associated processes. Aside the potential use of the PatchC]l[amper, we propose the use of RBCs from patients and genetically modified RBCs and to combine it with other biophysical methods like confocal live cell imaging, microfluidic approaches, and mathematical modeling. This research activity would require a level of patch-clamp mobility involving, e.g., the laboratories of all authors of this Speciality Grand Challenge. As a result we expect to understand the role of transient transmembrane cation transport in the dynamical behavior of RBCs in the circulation, and how this sustains the lifespan of in average 120 days.

### To Elucidate the Molecular and Functional Basis of Channelopathies in RBCs, as Well as the Secondary Involvement of Ion Channels in Congenital Anemia

We are only at the beginning to understand the role of ion channels as molecular regulators or modulators of RBC channelopathies and other RBC diseases, respectively. The availability of large patient cohorts allows to identify and characterize new genes involved in aberrant activation of ion channels, to clarify the pathogenetic mechanism of new variants identified in known genes and to verify the functional effects of the large number of “variants with unknown significance” (VUS) identified by next generation sequencing (NGS) approaches. A common treatment of hemolytic anemia is splenectomy. In hereditary xerocytosis (caused by a gain of function mutation in Piezo1), splenectomy results in a high incidence of thrombotic complications (Picard et al., 2019). One should make use of the available mouse model with a *Piezo1* gain of function mutation (Ma et al., 2018) to investigate the effect of splenectomy on the flow behavior of RBCs *in vivo*, and the thrombus formation in an experimental setup. The knowledge gained in the animal model should be used to further investigate blood samples of splenectomized patients to endorse a better patient treatment and disease management. This research activity would require a broad involvement of patients and the maximal regional operation of the PatchC]l[amper, i.e., whenever the patient/hospital can be reached on road within a reasonable effort. For individual patients this is within Europe, where the RBC community is traditionally well connected, e.g., through the EuroBloodNet initiative, the European Red Cell Society (ERCS) and numerous projects funded by the European Commission. For the involvement of patient groups, e.g., malaria, sickle cell disease, or thalassemia, the PatchC]l[amper could also move to other continents like Africa or Asia. For transoceanic investigations, a second PatchC]l[amper would probably being a preferred solution. Overall, we expect novel causative genes to be found involved in deregulated ion channel activity, and to have better insight in the role of ion channels in disease modulation including thrombus formation.

### To Assess the Role of Ion Channels During RBC Production (in Bioreactors)

We anticipate to bring automated high throughput patch-clamp recordings to the clinic and to the cell biology laboratories combined with the opportunity of bioreactor based *in vitro* erythropoiesis and the expertise in molecular biology to perform genetic manipulations. Recently, bioreactor based erythropoiesis progressed considerably (Hansen et al., 2019; Heshusius et al., 2019; Connor et al., 2021; Pellegrin et al., 2021), which showed the importance to understand channel activity (Piezo1) in this process (Aglialoro et al., 2021), especially since the cells in bioreactors are due to the nature of these reactors under constant flow or shear stress. Erythropoiesis under physiological conditions in the bone marrow occurs rather in the absence of flow, but crawling over macrophages may cause shear stress. Several groups work on the further improvement of bioreactor technology. The goal is to decrease the costs with the clear aim to allow the use of *in vitro* (designed) RBCs in transfusion medicine.

All these developments enable a unique systematic approach to identify and understand the role of ion channels during maturation and function of RBCs in health and disease. To this end, it is advisable to compare patient RBCs with known mutations with their counterparts generated *in vitro* to understand the ion channel contribution during cellular development and to create treatment concepts based on that knowledge. The inclusion of multi-omics offers the possibility to identify cellular processes controlled by ion fluxes and membrane potential. One can in principle test the physical and biological effect of novel mutations and one can explore how to control ion channel activity during the large scale production of cultured RBCs for transfusion purposes in a stirred bioreactor. There is the option to characterize ion channel activity during terminal differentiation of RBC precursors to mature, functional cells. Furthermore, we have the opportunity to confirm or negate the role of VUS in ion channels detected by NGS in anemia patients.

## Summary

The research concept described above enables a unique systematic approach to identify and understand the role of ion channels during maturation and function of RBCs in health and disease and we expect to understand how ion channel activity has to be regulated during the production of cultured blood to obtain functional RBCs for transfusion purposes.

## Author Contributions

ML, SE, PB, and LK jointly wrote the manuscript and agreed to be accountable for the content of the work. All authors contributed to the article and approved the submitted version.

## Conflict of Interest

The authors declare that the research was conducted in the absence of any commercial or financial relationships that could be construed as a potential conflict of interest.

## Publisher’s Note

All claims expressed in this article are solely those of the authors and do not necessarily represent those of their affiliated organizations, or those of the publisher, the editors and the reviewers. Any product that may be evaluated in this article, or claim that may be made by its manufacturer, is not guaranteed or endorsed by the publisher.
